# Prokineticin 2 Upregulation in the Peripheral Nervous System Has a Major Role in Triggering and Maintaining Neuropathic Pain in the Chronic Constriction Injury Model

**DOI:** 10.1155/2015/301292

**Published:** 2015-01-05

**Authors:** Roberta Lattanzi, Daniela Maftei, Veronica Marconi, Fulvio Florenzano, Silvia Franchi, Elisa Borsani, Luigi Fabrizio Rodella, Gianfranco Balboni, Severo Salvadori, Paola Sacerdote, Lucia Negri

**Affiliations:** ^1^Department of Physiology and Pharmacology “Vittorio Erspamer”, Sapienza University of Rome, 00185 Rome, Italy; ^2^Confocal Microscopy Unit, European Brain Research Institute (EBRI) “Rita Levi-Montalcini”, 00143 Rome, Italy; ^3^Department of Pharmacological and Biomolecular Sciences, University of Milan, 20129 Milan, Italy; ^4^Unit of Human Anatomy, Department of Biomedical Sciences and Biotechnologies, University of Brescia, 25123 Brescia, Italy; ^5^Department of Life and Environment Sciences, University of Cagliari, 09124 Cagliari, Italy; ^6^Department of Chemical and Pharmaceutical Sciences, University of Ferrara, 44-100 Ferrara, Italy

## Abstract

The new chemokine Prokineticin 2 (PROK2) and its receptors (PKR_1_ and PKR_2_) have a role in inflammatory pain and immunomodulation. Here we identified PROK2 as a critical mediator of neuropathic pain in the chronic constriction injury (CCI) of the sciatic nerve in mice and demonstrated that blocking the prokineticin receptors with two PKR_1_-preferring antagonists (PC1 and PC7) reduces pain and nerve damage. PROK2 mRNA expression was upregulated in the injured nerve since day 3 post injury (dpi) and in the ipsilateral DRG since 6 dpi. PROK2 protein overexpression was evident in Schwann Cells, infiltrating macrophages and axons in the peripheral nerve and in the neuronal bodies and some satellite cells in the DRG. Therapeutic treatment of neuropathic mice with the PKR-antagonist, PC1, impaired the PROK2 upregulation and signalling. This fact, besides alleviating pain, brought down the burden of proinflammatory cytokines in the damaged nerve and prompted an anti-inflammatory repair program. Such a treatment also reduced intraneural oedema and axon degeneration as demonstrated by the physiological skin innervation and thickness conserved in CCI-PC1 mice. These findings suggest that PROK2 plays a crucial role in neuropathic pain and might represent a novel target of treatment for this disease.

## 1. Introduction

Identification of the neurobiological processes engaged in the pathological state that occurs during neuropathic pain may provide future therapeutic targets. Chemokines and their receptors are receiving growing interest as modulators of neuronal plasticity and for their ability to enhance nociceptive transmission under conditions of neuropathic pain [1]. In particular a new family of chemokines, Prokineticin 2 (PROK2) also known as Bv8 and its receptors, two G-protein coupled receptors, PKR_1_ and PKR_2_, emerged as key signals in immune system and inflammatory diseases [2].

In an animal model of CFA-induced paw inflammation, we brought evidence that Bv8/PROK2, upregulated in granulocyte invading the inflamed tissue is a major determinant in triggering and maintaining inflammatory pain [3]. Neutrophils and macrophages are the major sources of PROK2 which is strongly upregulated in inflammatory diseases and tumours, associated with infiltrating cells [4, 5].* In vivo *and* in vitro* experiments from our and other groups demonstrated potent chemotactic and immunomodulatory activities of the prokineticins, able to promote the production of proinflammatory cytokines from different immune cells and to modulate T cell function by reducing anti-inflammatory cytokines production and promoting Th1 responses [5–7].

The typical immune cell response to tissue injury is largely conserved in the lesioned peripheral nervous system: many neutrophils from the circulation invade the area immediately around the nerve injury site within 8–24 hours and haematogenous macrophages by 3-4 days post injury, whereas lymphocyte accumulation in the injured nerve is delayed by a week or more [8].

We can reasonably presume that PROK2 released by haematogenous neutrophils invading the damaged nerve triggers inflammation and contributes to pain.

Besides, in the immune system, PROK2 is also expressed in discrete nuclei into the brain and is constitutively expressed, at very low levels, in some DRG neurons also expressing the vanilloid receptor TRPV1 [9–11]. PROK2 binds PKR_1_ and PKR_2_, expressed in small and medium cells of rat dorsal root ganglia (DRG) and within the superficial laminae in the spinal dorsal horn [2, 12]. Peripheral administration of the mammalian PROK2 or its amphibian homologue Bv8 in rodents induced hyperalgesia and allodynia by activating the PKRs on peripheral nociceptors.* In vitro* analysis of functional PKRs on DRG neurons shows a remarkable overlap in neurons that respond both to Bv8 and capsaicin (~90%) and to Bv8 and mustard oil (~60%) indicating a high degree of colocalization of functional PKRs with TRPV1 and TRPA1 channels [13] supporting the possibility that these receptors contribute to nociceptive signalling. Moreover Bv8, increasing CGRP and SP expression, induced long-lasting sensitization of nociceptors resulting in enhanced responses to evoking stimuli, including Bv8 itself or capsaicin, so providing a basis for long-lasting hypersensitivity that may result in conditions of tissue injury [14].

We have recently demonstrated in an animal model of neuropathic pain, the chronic constriction injury of the sciatic nerve (CCI), that 10 days after nerve injury an important activation of the prokineticin system is evident at peripheral and central level and that pharmacological blocking of the prokineticin receptors with the antagonist PC1 abolishes pain and controls some pathophysiological processes underlying the neuropathy.

Here we analysed the time-course of PROK2 upregulation in the DRG and in the injured sciatic nerve of mice repeatedly treated with saline or with PC1 for 7 days and demonstrated that the antihyperalgesic effect of PC1 temporally correlates with its ability to reduce the neuropathy-induced increase of PROK2 expression. The recent availability of a new prokineticin receptor antagonist, PC7, endowed with higher affinity and selectivity for the PKR1 pushed us to evaluate its antihyperalgesic effect in comparison with that of PC1 [15, 16]. Finally, because CCI of sciatic nerve decreases the density of paw skin innervation and induces epidermal thinning we also compared the fibre-density in the dermis and the skin thickness of the injured paw of mice treated with PC1 or with saline in comparison with sham operated mice.

## 2. Methods

### 2.1. Animal Preparation

Experiments were carried out in male CD1 mice (25–30 g, Harlan Laboratories, Italy) according to protocols approved by the Animal Care and Use Committee of the Italian Ministry of Health and in compliance with the IASP and European Community (E.C.L358/118/12/86) guidelines. All efforts were made to minimize animal suffering and to reduce the number of animals used. Animals were housed individually in cages, under conditions of optimum light, temperature, and humidity (12 : 12 h light/dark cycles, 22± 2°C, 50–60%) with food and water ad libitum and acclimatized to the environment for 4-5 days before surgery or pharmacologic treatment.

### 2.2. Neuropathic Pain Model

#### 2.2.1. Chronic Constriction Injury of the Sciatic Nerve (CCI)

Mononeuropathy was induced by the CCI of the sciatic nerve [17] in CD1 mice anesthetized by intraperitoneal injection of ketamine-xylazine (60 mg kg^−1^ + 10 mg kg^−1^). The sciatic nerve was exposed and three loose ligatures with 4–0 silk suture thread were made around the nerve with a 1.0–1.5 mm interval between each of them. In sham-operated mice, an identical dissection was performed on the same side, except that the sciatic nerve was not tied.

### 2.3. Nociceptive Behavioural Tests

Behavioural experiments were carried out by researchers blind to treatments, between 10 am and 2 pm, in a reserved quiet temperature-controlled room. Mice were habituated to the testing environment and were handled daily two times for at least three days before baseline testing.

For testing heat sensitivity, animals were put in plastic boxes and allowed 30 min for habituation before examination. Heat sensitivity was tested by radiant heat using Hargreaves apparatus (Ugo Basile, Italy) and expressed as paw withdrawal latency (PWL). The radiant heat intensity is adjusted so that basal PWL is between 10 and 12 s with a cutoff of 20 s to prevent tissue damage. For testing mechanical sensitivity, animals were put in boxes on an elevated metal mesh floor and allowed 30 min for habituation before examination. Mechanical allodynia was assessed using the Dynamic Plantar Aesthesiometer (Ugo Basile Italy). The filament was applied to the skin of the midplantar area of the hind paw, and it began to exert an increasing upward force, reaching a maximum of 30 g in 10 s, until the paw was withdrawn. The paw withdrawal threshold (PWT) was defined as the force, in grams, at which the mouse withdrew its paw. PWL and PWT of ipsilateral and contralateral paws were measured thrice, and the reported value is the mean of the three evaluations.

### 2.4. Experimental Design

The PKR antagonists PC1 [16] and PC7 were injected in mice subcutaneously (s.c., 50 *μ*L for 10 g body weight) into the flank region of the mouse.

PC1 was administrated chronically at the dose of 150 *μ*g kg^−1^, s.c., twice a day for 7 days.

Increasing doses of PC7 (5, 15, and 45 *μ*g kg^−1^) were administrated s.c. as a single bolus in different groups of mice, on day 3 post injury (dpi). Then we chose the highest, more effective dose (45 *μ*g kg^−1^ s.c.) for the repeated treatment, twice a day for 4 days.

PC7 (2-(5-(4-fluorobenzyl)-1-(4-methoxybenzyl)-1,4,5,6-tetrahydro-4,6-dioxo-1,3,5-triazin-2-ylamino)-ethyl)-guanidine) is a triazinic compound which displays higher affinity for the PKR_1 _(IC_50_ = 56 ± 12 nM, in displacement of ^125^I-MIT; IC_50_ = 66 ± 12 nM in BRET assay) than for PKR_2_ (IC_50_ = 5230 ± 700 nM in displacement of ^125^I-MIT; IC_50_ = 4135 ± 600 nM in BRET assay) and is able to antagonize the Bv8-induced hyperalgesia at doses ten times lower than PC1 [15].

Mice were divided as follows: (i) CCI mice treated with saline (CCI-saline, *n* = 8); (ii) CCI mice treated with PC1 150 *μ*g kg^−1^ s.c., twice a day for 7 days or PC7 45 *μ*g kg^−1^ s.c., twice a day for 4 days, starting from day 3 after CCI (CCI-PC1, CCI-PC7, *n* = 8/group); (iii) Sham-operated mice treated with saline (*n* = 5); (iv) Sham-operated mice treated with PC1 150 *μ*g kg^−1^ s.c., twice a day for 7 days or PC7 45 *μ*g kg^−1^ s.c., twice a day for 4 days, starting from day 3 after sham surgery (*n* = 5/group) (data not shown).

In sham, CCI-saline, and CCI-PC1 or CCI-PC7 animals, mechanical allodynia and thermal hyperalgesia were assessed before and from days 1 to 12 after CCI.

### 2.5. Biochemical and Histochemical Evaluation

#### 2.5.1. RNA Extraction and Real-Time PCR

Total RNA was extracted from L4-L5 DRG and sciatic nerve, pooled from two mice, using the Trizol reagent (Invitrogen, Carlsbad, CA) according to the manufacturer's instruction. RNA yield and purity were determined by spectrophotometry absorption at 260 and 280 nm. To obtain cDNA, 1 *μ*g of mRNA underwent to Reverse Transcription (Promega, Madison, WI). The resulting cDNA was stored at −20°C until used for the further analysis. Messenger RNA expression was quantitatively measured with quantitative (q) real time PCR using iCycler Bio-Rad. The reaction was performed in a 25 *μ*L volume using SensiMix SYBR Green & Fluorescein kit (Bioline, London, UK). All the measures were performed in triplicate. The reaction conditions were as follows: 95°C for 10 min (Polymerase activation), followed by 40 cycles at 95°C for 15 min, 55–50°C (temp. depends on the Tm of primers) for 15 sec and 72°C for 15 sec. The reaction mixture without the cDNA was used as control. The primer sequences used in this study were as follows for PROK2: forward 5′-CTCGGAAAGTTCCATTTTGG-3′ and reverse 5′-TTCCGGGCCAAGCAAATAAACC-3′, Glyceralde-hydes-3-phosphate dehydrogenase (GAPDH): forward 5′-GCCAAGGCTGTGGGCAAGGT-3′ and reverse 5′-TCTCCAGGCGGCACGTCAGA-3′. The Ct value of the specific gene of interest was normalized to the Ct value of the endogenous control, GAPDH, and the comparative Ct method (2-ΔΔCt) was then applied using sham mice group as calibrator. Results are mean ± SEM of at least 3 experiments.

#### 2.5.2. Cytokine Protein Measurement

Ten days after injury nerve samples were homogenized in ice-cold phosphate-buffered saline containing a protease inhibitor cocktail (Roche Diagnostics, Monza, Italy). IL-6, IL-1*β*, IL-10 TNF*α*, and IL-17 protein contents were determined by a multiplex enzyme-linked immunosorbent assay using ultrasensitive murine ELISA (Milliplex, Millipore, Vimodrone, Italy). A standard curve ranging on average from 0.15 pg/mL to 3700 pg/mL was prepared and then fitted by Bio-Plex Manager software.

#### 2.5.3. Immunofluorescence

At 10 dpi DRG, sciatic nerve and plantar skin were dissected from transcardially perfused (PBS followed by 4% paraformaldehyde) mice embedded in cryostat medium and frozen. Serial sections (20 *μ*m) were cut using a cryostat and thaw-mounted onto glass slides. Prior to immunofluorescence staining all sections were blocked with 3% normal donkey serum (serum source was the same as the secondary antibody producing species), containing 0.3% Triton X-100 for 1 h at room temperature. Then the sections were incubated at 4°C overnight with the following primary antibodies diluted in PBS-0.3% Triton X-100: 1/200 rabbit polyclonal anti-PROK2 (AbCam, Cambridge, UK), 1/400 mouse polyclonal anti-glial fibrillary acidic protein (GFAP) (Immunological Sciences, Italy), 1/100 rat monoclonal anti-CD11b (BD Pharmigen, Italy), 1/300 mouse monoclonal anti-S100*β* (Sigma-Aldrich, Milano, Italy), 1/200 mouse monoclonal [NF-200] to hypophosphorylated neurofilament H (AbCam, Cambridge, UK), and 1/300 goat polyclonal anti-CGRP (AbCam, Cambridge, UK). The sections were then incubated for 2 h at room temperature in 1 : 200 anti-species IgG antibodies coupled to Alexa Fluor-488 or 555 (Immunological Sciences, Italy). Nuclei were stained with DAPI 1/500. The stained sections were examined at confocal laser scanning microscope (Leica SP5, Leica Microsystems, Germany). Immunofluorescence intensity or immunoreactive area was measured in five fields (300 *μ*m^2^) for every section in at least 10 sections for every experimental group (http://imagej.nih.gov/ij/index.html free software).

The specificity of the anti-PROK2 antibody was tested preadsorbing it with the protein PROK2 (500 ng) overnight at 4°C before incubation with tissue.

#### 2.5.4. Plantar Skin Histology

At 10 dpi the skin samples from plantar surface of sham, CCI-saline, and CCI-PC1 mice were embedded in paraffin in a correct orientation, so that they could be sectioned perpendicular to the skin surface.Plantar skin sections (5 *μ*m) were deparaffinized, hydrated and stained by a brief immersion in Mayer's hematoxylin and eosin, followed by a brief dehydration in ethanol and xylene, and mounted with DPX mounting medium (Sigma, St. Louis, MO, USA). Sections were photographed at 20x magnification (Olympus U-CMAD3, Japan). Epidermal thickness, indicated as the distance between the dermoepidermal junction and the top of the outer most granular layer, was evaluated in three sections per tissue sample. On each image 4-5 measurements were made along the strip of epidermis to assess the average thickness. Data were expressed as mean ± SEM.

### 2.6. Evans Blue Assay

The permeability of blood nerve barrier (BNB) was determined by Evans Blue dye extravasation in sciatic nerve, 3 dpi. Evans Blue dye (4%, 5 mL/kg) was injected into the tail vein of anaesthetized mice. After 30 min animals were perfused with PBS. The sciatic nerves were removed and incubated in 1 mL formamide (Sigma-Aldrich) at 60°C for 24 h. Evan's Blue concentration was determined using a spectrophotometer (Shimadzu UV-160A) at a wavelength of 620 nm.

The data are expressed as *μ*g of Evans Blue per g of tissue.

### 2.7. Statistic

Results are expressed as mean ± SEM. When appropriate, One-way ANOVA followed by Tukey's posttest for multiple comparisons or Two-way ANOVA followed by Bonferroni's posttest repeated measures were performed using GraphPad Prism 5 for Windows version 5.4. Differences were considered significant at ^∗°^ *P* < 0.05, ^∗∗°°^ *P* < 0.01, and ^∗∗∗°°°^ *P* < 0.001.

## 3. Results and Discussion

### 3.1. Nociceptive Behavioural Results

**Figure 1:**
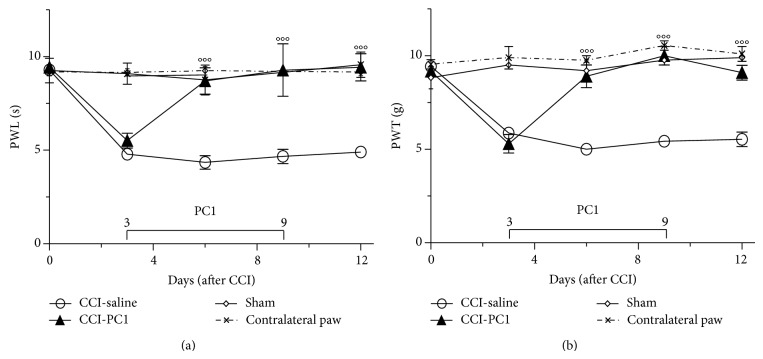
Repeated systemic injections of PC1 (150 μg kg^−1^, twice a day) from 3 to 9 dpi reverted the CCI-induced thermal hyperalgesia (a) and mechanical allodynia (b) in two days. The antihyperalgesic effect lasted after treatment withdrawal, for all the observation period. Data represent means ± SEM of 6–9 mice. Two-way ANOVA was used for statistical evaluation, followed by Bonferroni's test. °°°*P* < 0.001 CCI-PC1 versus CCI-saline mice.

Figure 1 shows that, since day 3 after CCI, thermal and mechanical nociceptive thresholds decrease of 40%–50% in the ipsilateral compared to contralateral hind paw. Both allodynia and thermal hyperalgesia were observed at the following postlesion time points: 3, 6, 10, and 12 days.

Therapeutic treatment of CCI mice with PC1 from day 3 (when hyperalgesia peaks) to day 9 post injury (dpi) abolished thermal and also mechanical hyperalgesia so that from 6 up to 12 dpi PWL and PWT of injured paw did not differ from those of sham mice (Figure 1).

**Figure 2:**
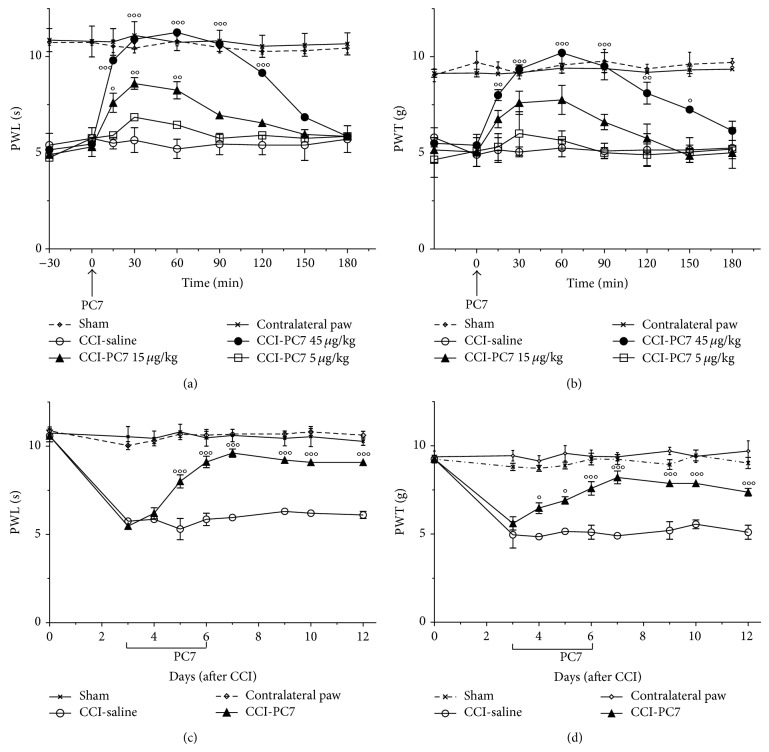
Antihyperalgesic effect of PC7. A single bolus s.c. injection of PC7 (5, 15, and 45 μg kg^−1^) on day 3 after CCI dose-dependently reverted the established CCI-induced thermal hyperalgesia (a) and mechanical allodynia (b). The highest dose of PC7 (45 μg kg^−1^) abolished hyperalgesia for about 3 h. Repeated systemic injections of PC7 (45 μg kg^−1^, twice a day) from 3 to 7 dpi significantly reduced the CCI-induced thermal hyperalgesia (c) and mechanical allodynia (d) for all the observation period. Data represent means ± SEM of 5 mice. Two-way ANOVA was used for statistical evaluation, followed by Bonferroni's test. °*P* < 0.05; °°*P* < 0.01; °°°*P* < 0.001 CCI-PC7 versus CCI-saline mice.

Availability of a new Bv8 antagonist, named PC7, able to antagonize the Bv8-induced hyperalgesia at doses ten times lower than PC1 and endowed with higher selectivity for the PKR_1_ [15] pushed us to evaluate its efficacy in this neuropathic pain model. Single bolus s.c. injections of PC7 at 3 dpi, when thermal and mechanical hyperalgesia reached full development, dose-dependently reduced pain (Figures 2(a), and 2(b)) at doses (5, 15, and 45 *μ*g kg^−1^) about 3 times lower than the doses of PC1 which we have already demonstrated able to reduce/abolish the CCI-induced thermal and tactile hyperalgesia [16]. The antihyperalgesic effect of the highest dose (45 *μ*g kg^−1^, s.c.) peaked in 15 minutes and lasted for 3 h. Then we treated the mice with the highest dose (45 *μ*g kg^−1^, s.c) two times/day for only four days (3 to 6 dpi) and measured the nociceptive threshold every morning up to 12 dpi. Like PC1, PC7-treatment abolished the nerve-injury-induced thermal and mechanical hyperalgesia within 2 days and continued to confer protection over the observation period, up to 12 dpi (Figures 2(c), and 2(d)). Repeated administration of PC1 or PC7 did not change the thermal and mechanical nociceptive thresholds of sham mice (data not shown).

The prompt reversal of pain after acute administration depends on blocking the PKRs on peripheral sensory neurons. Indeed, as we have already demonstrated both receptors mediate the Bv8/PROK2-induced decrease of nociceptive threshold: PKR_1_ in cooperation with TRPV1 is the receptor mainly responsible for thermal hyperalgesia and PKR_2_ mainly contributes to mechanical allodynia [16].

### 3.2. PROK2 mRNA Time-Course in DRG and Sciatic Nerve

In a previous paper we have already demonstrated that at 10 dpi PROK2 was overexpressed in the periphery and in the spinal cord of neuropathic mice but was maintained close to physiological levels in neuropathic mice treated with PC1 for 7 days.

**Figure 3:**
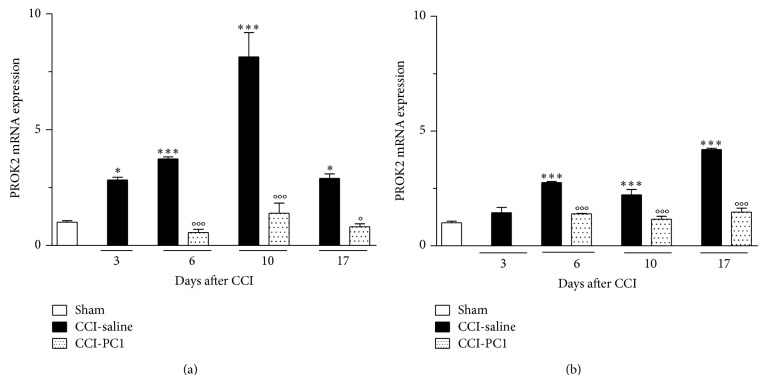
Time-course of PROK2 mRNA expression in injured sciatic nerve and ipsilateral DRG of CCI-saline mice and CCI-PC1 mice. PROK2 levels in the peripheral nervous system of healthy animals were negligible (33.49 ± 0.09 Ct in sciatic nerve, 33.97 ± 0.41 Ct in DRG). In the sciatic nerve (a) PROK2 expression was significantly increased at 3 dpi, reached its maximal expression at 10 dpi, and then started to decrease up to 17 dpi. In the ipsilateral L 4–6 DRGs (b) PROK2 mRNA was significantly increased at 6 dpi and showed a constant tendency to increase up to 17 dpi. Data are mean ± SEM of 5 animals. One-way ANOVA was used for statistical evaluation, followed by Tukey test for multiple comparisons. ^*^ *P* < 0.05, ^***^ *P* < 0.001 CCI-saline versus sham; °*P* < 0.05 CCI-PC1 versus CCI-saline.

Here we studied in detail the time-course (from day 1 to 17) of injury-induced PROK2 mRNA overexpression in the peripheral nervous system. PROK2 levels in the peripheral nervous system of healthy animals were negligible. RT-PCR gave 33.49 ± 0.09 Ct in the sciatic nerve and 33.97 ± 0.41 Ct in the DRG. However, as reported in Figure 3(a), in the sciatic nerve the PROK2 expression was significantly increased at 3 dpi (about 3-fold) when thermal and mechanical hyperalgesia were already evident, reached its maximal expression (8 fold) at 10 dpi, and then started to decrease up to 17 dpi. In the ipsilateral L4-6 DRG it started to increase later, at 6 dpi, and showed a constant tendency to increase up to 17 dpi (Figure 3(b)). The fact that PROK2 overexpression in DRG was delayed and lasted longer than in sciatic nerve indicates the presence of a flow of activation that, starting from the periphery, moves towards the centre. The early increase of PROK2 expression in the injured nerve is probably associated with neutrophils and Schwann Cells (SC) which express PROK2 (see afterwards) and which begin to dedifferentiate and to proliferate within 48 h of injury reaching the peak of proliferation around 4 dpi [8]. Given that PROK2 promotes survival and migration of granulocytic and monocytic lineages, the injury induced increase of PROK2 in the nerve might activate PKR_1_ expressed on different immune cells thus promoting the further recruitment of new ones. Infiltration of macrophages rich in PROK2 together with the subsequent PROK2 upregulation in the nerve (see afterwards) answers for the further increase in PROK2 mRNA that gets to maximum level in the nerve at 10 dpi. The PROK2 mRNA levels in the sciatic nerve and DRG of PC1 treated mice did not differ from the PROK2 levels of sham animals at any time points.

Then we performed a detailed analysis of the cellular localization and modulation of PROK2 in the peripheral nervous system at 10 dpi, the time of its maximal expression.

### 3.3. PROK2 Localization in Dorsal Root Ganglia

**Figure 4:**
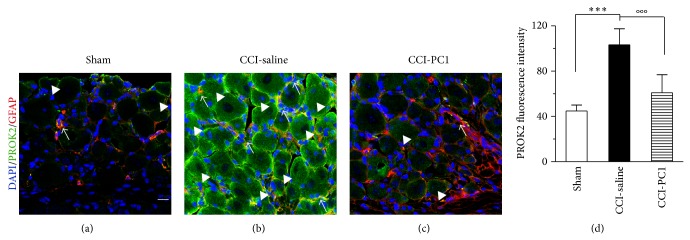
Representative sections of mouse L4-L5 ipsilateral DRG, at 10 dpi, from sham (a), CCI-saline (b), and CCI-PC1 (c) mice. Immunofluorescence double-staining of PROK2 (green) with GFAP (marker for satellite cells, red). Cell nuclei were counterstained with DAPI (blue fluorescence). In DRG of sham-operated mice the PROK2 signal was very faint, localized only along cell membrane of some neurons, mainly small sized (arrowheads), and in few GFAP+ satellite cells (arrow) (a). In neurons of CCI-saline mice, PROK2 immunofluorescence was significantly increased and showed a vesicular cytoplasmatic pattern which is dense in proximity of the neuronal membrane (arrowheads). The number of PROK2+ satellite cells is increased (arrows). PROK2 signal in CCI-PC1 mice was comparable with that of sham mice (c). Scale bar, 30 μm. (d) Evaluation of PROK2 fluorescence intensity. One-way ANOVA was used for statistical evaluation, followed by Tukey test for multiple comparisons ^***^ *P* < 0.001 CCI-saline versus sham mice; °°°*P* < 0.001 CCI-PC1 versus CCI-saline mice.

At 10 dpi, immunohistochemical analysis demonstrated a strong increase of immunoreactive protein PROK2 in the ipsilateral DRG of neuropathic mice respect to DRG of sham-operated mice, where the PROK2 signal was very faint (Figures 4(a), and 4(b)). Moreover in the ipsilateral DRG, the number of PROK2 positive neurons was frankly increased respect to sham mice, with immunofluorescence distributed in the whole cell body. PROK2 showed a cytoplasmatic vesicular pattern, characteristic of proteins that are packaged and transported/released. PROK2 fluorescence was also increased in some GFAP positive cells around the neuronal bodies (Figure 4, arrows) indicating that PROK2 is expressed also by satellite cells. In PC1-treated mice PROK2 signal was significantly reduced mainly in neurons, compared to the saline-treated neuropathic mice (Figure 4(c)) as demonstrated by comparison of PROK2 immunofluorescence intensity in sham, CCI-saline, and CCI-PC1 mice (Figure 4(d)).

### 3.4. PROK2 Localization in Sciatic Nerve

**Figure 5:**
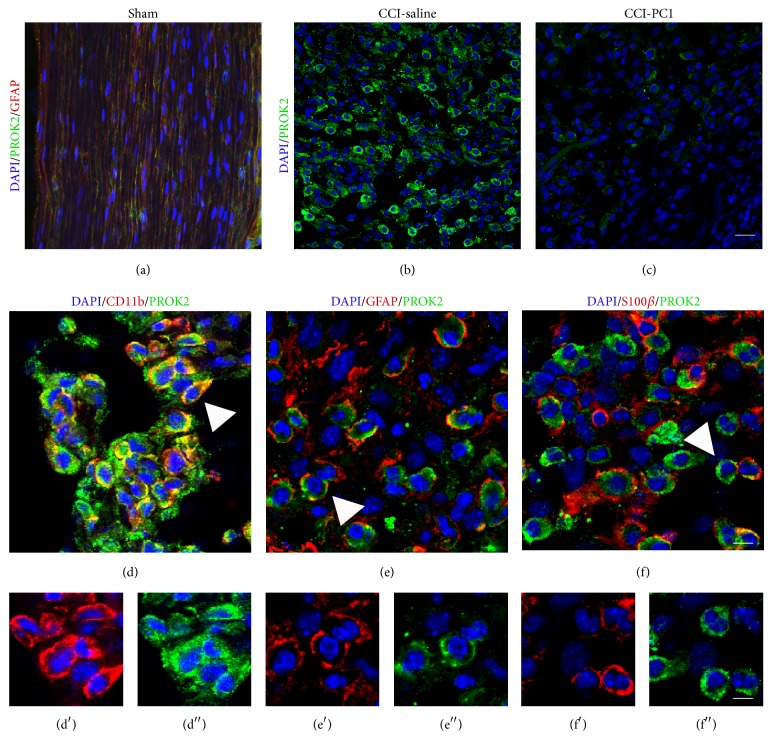
Representative images of sciatic nerve section in the immediate proximity of the injury, from sham (a), CCI-saline (b), and CCI-PC1 (c) mice at 10 dpi. (a) In the sciatic nerve of sham-operated mice PROK2 immunoreactivity (green) was very faint and colocalized with GFAP (red) in elongated SC. (b) A heavy infiltration of PROK2-positive cells (green) was evident in the nerve from CCI-saline mice. (c) PC1 treatment significantly reduced the PROK2 immunoreactivity in these cells. Scale bar, 30 μm. Immunofluorescence double-staining showing colocalization (yellow, arrowheads) of PROK2 (green) with CD11b (macrophage marker, red) (d), GFAP (Schwann cell marker, red) (e), and S100β (Schwann cell marker, red) (f) in the immediate proximity of the injury in the sciatic nerve of CCI-saline mice. (d′) CD11b (red), (e′) GFAP (red), (f′) S100β (red), and (d′′, e′′, f′′) PROK2 (green) shown in single channels. Scale bar, 10 μm. Cell nuclei were counterstained with DAPI (blue fluorescence).

**Figure 6:**
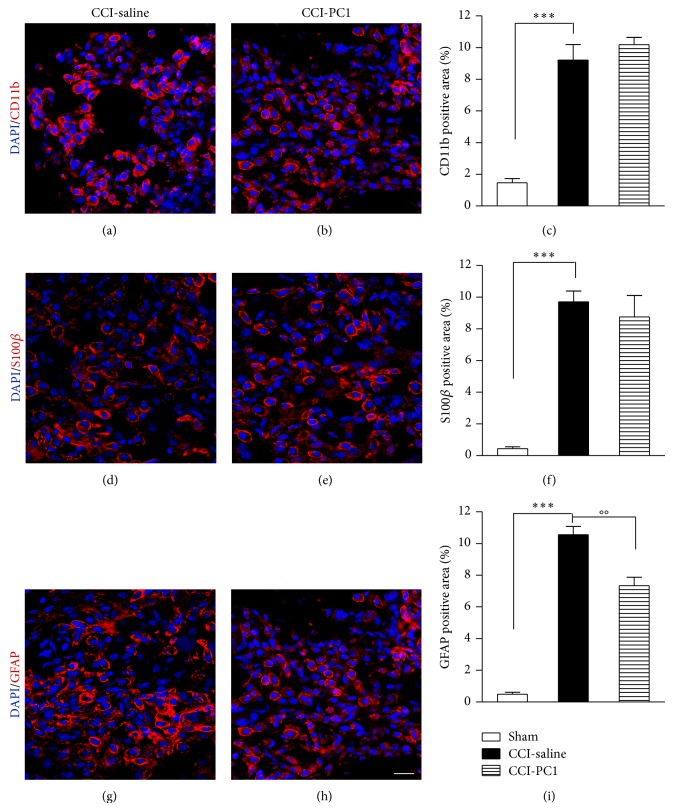
Immunostaining of activated macrophages (CD11b+, red), S100β+ SC (red), and GFAP+ SC (red) in the neuroma from CCI-saline and CCI-PC1 mice ((a)–(h)). Repeated treatment with the PKR-antagonist significantly reduced the GFAP+ activated SC (i) but did not affect S100β+ SC or macrophage infiltration ((c), (f)).

In the sciatic nerve of sham-operated mice PROK2 immunoreactivity (green) was very faint and appeared colocalized in GFAP positive cells (yellow) (Figure 5(a)) whereas on 10 dpi a heavy infiltration of PROK2-positive cells (green colour, Figure 5(b)) was evident in the neuroma in the immediate proximity of the injury. The PROK2 positive cells are macrophages as demonstrated, in Figure 5(d), by double staining (yellow) with the macrophage marker CD11b (red). But they are also activated Schwann cells (SC) GFAP-positive (Figure 5(e)) and myelinating SC identified by S100*β* (Figure 5(f)). One week repeated treatment with the PKR-antagonist dramatically reduced the PROK2 immunofluorescence but not the number of infiltrating cells (Figure 5(c)). However, comparing the percent CD11b-, GFAP-, and S100*β*-positive area in injured nerve from saline-treated mice with respect to injured nerve from PC1-treated mice showed that the %GFAP-positive area was significantly reduced by PC1-treatment suggesting a lesser activation of SC whereas the pharmacological treatment had not affected macrophage recruitment nor myelinating SC (Figures 6(a)–6(i)). Lesser activation of SC may be a proof of lesser axonal degeneration in PC1-treated animals.

**Figure 7:**
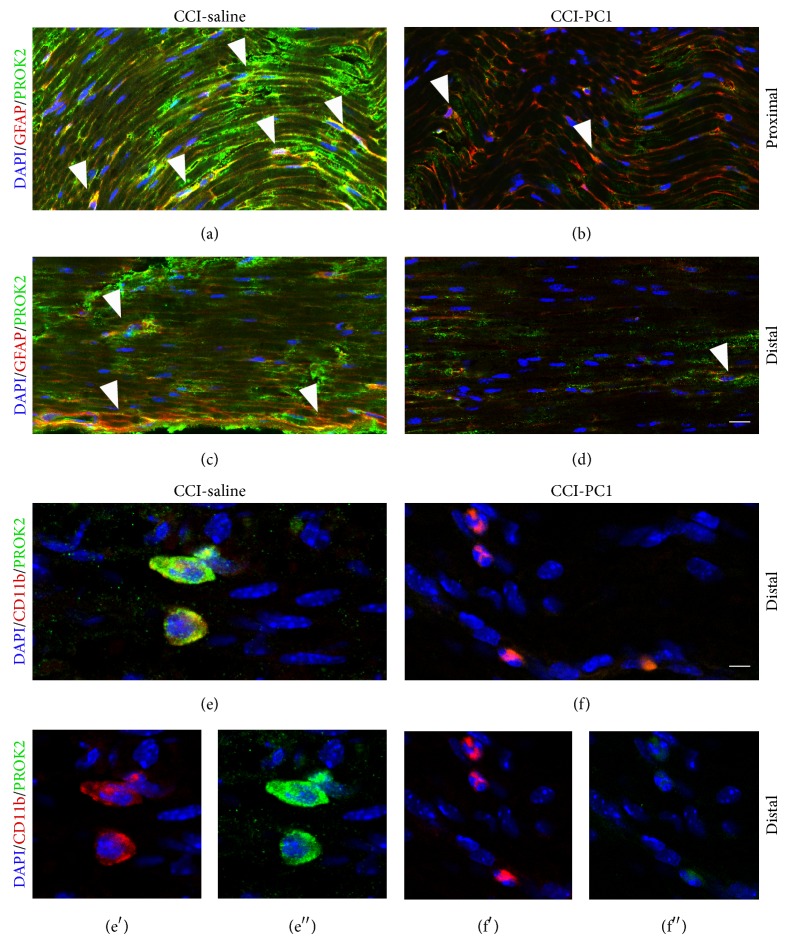
Representative images of CCI-induced upregulation of PROK2 in the longitudinally sliced sciatic nerve proximal and distal to the lesion. At 10 dpi a dramatic increase of PROK2 signal (green, a and c) in fibres and in GFAP+ structures (yellow) was evident both proximal and distal to the lesion. The PROK2 signal was dramatically reduced in the nerve from CCI-PC1 mice (green, b and d). Scale bar: 30 μm. High-magnification images showed macrophages that infiltrate the nerve distal to the lesion (scale bar: 10 μm). (e) Double immunofluorescence labelling for PROK2 (green) and CD11b (red) showing that in the CCI-saline mice the infiltrating macrophages contain PROK2 (yellow). (f) PROK2 signal was absent in macrophages infiltrating the nerve from CCI-PC1 mice. (e′, f′) CD11b (red), and (e′′, f′′) PROK2 (green) shown in single channels. Cell nuclei were counterstained with DAPI (blue fluorescence).

**Figure 8:**
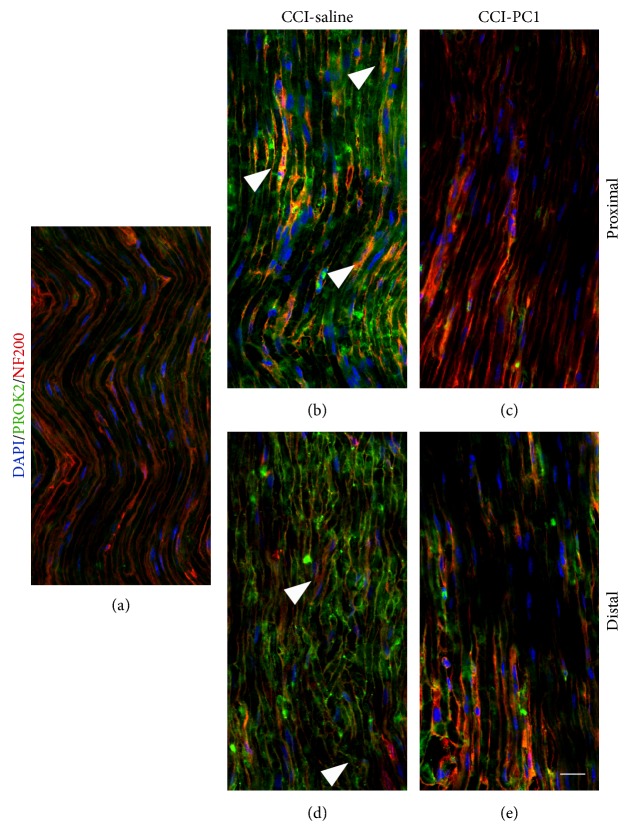
Confocal images of representative sections of longitudinally sliced sciatic nerve, proximal and distal to the lesion, immunostained for PROK2 (green) and NF200 (red) from sham-operated, CCI-saline and CCI-PC1 mice at 10 dpi. Scale bar: 30 μm. PROK2-green signal was localized between NF200 positive fibers in CCI-saline mice but was not found in the nerve from CCI-PC1 mice.

**Figure 9:**
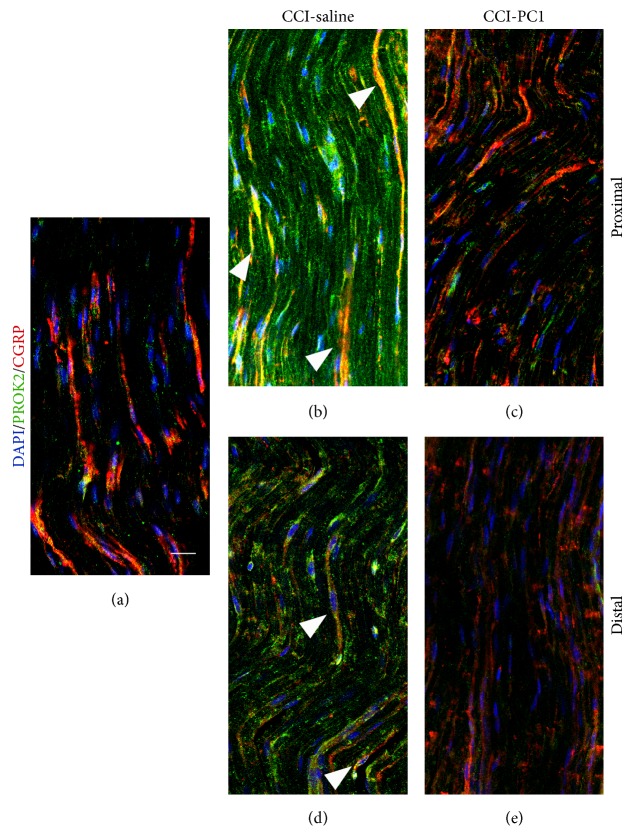
Confocal images of representative sections of longitudinally sliced sciatic nerve proximal and distal to the lesion, immunostained for PROK2 (green) and CGRP (red) from sham-operated, CCI-saline and CCI-PC1 mice at 10 dpi. Scale bar: 30 μm.

In longitudinal sections of the sciatic nerve (Figures 7, 8, and 9), immunofluorescence staining of nerve proximal and distal to the lesion demonstrated a dramatic increase of PROK2 signal (green) in the endoneurial space. The green signal was distributed in elongated structures, probably axons, and appeared more intense in the proximal than distal nerve suggesting that it is transported from DRG towards the peripheral terminals.

Double staining with GFAP demonstrated PROK2 in SC (yellow) scattered between fibres in the proximal nerve (Figure 7(a)) and mainly aligned to form a band in the distal nerve (Figure 7(c)), probably the bands of Büngner, which provide a supportive substrate and growth factors for regenerating axons [18]. In the injured sciatic nerve from PC1-treated mice the PROK2 immunofluorescence was dramatically reduced both in the fibres and in the SC (Figures 7(b) and 7(d)).

In the distal but not proximal injured nerve we found large CD11b+ cells many of which are also positive for PROK2 in neuropathic mice (Figure 7(e)), whereas the CD11b+ cells did not show any costaining with PROK2 in PC1-treated neuropathic mice (Figure 7(f)), confirming that PC1 treatment prevented the PROK2 upregulation also in macrophages.

In CCI-mice double-stained with NF200, which recognizes the heavy chain of neurofilaments

in myelinated fibres [19], strong PROK2-green signal was localized between NF200 positive fibres. This staining was not found in the nerve of PC1-treated mice (Figures 8(a)–8(e)).

Double staining with CGRP, which recognizes peptidergic neurons, demonstrated the presence of PROK2 protein in CGRP-immunoreactive (IR) fibres in proximal and in distal injured nerve of saline treated mice. PROK2 signal was very low in injured nerve of PC1-treated mice (Figures 9(a)–9(e)).

These analyses clearly demonstrate that during nerve injury a large amount of PROK2 is expressed by almost all cell types present in the nerve, both resident and infiltrating, for a sustained period of time.

Bv8/PROK2-PKRs are therefore ligand/receptor pairs in the regulation of pain sensation [20, 21] and the transient receptor potential vanilloid 1 (TRPV1) is a critical molecular link between PKRs and primary sensory neuron activation [13]. TRPV1 is upregulated by nerve injury and TRPV1, as well as the PKRs, is also expressed in sensory nerve axons of peripheral nerves, not only at peripheral terminal [16, 22]. It is quite possible, therefore, that axonal TRPV1, just like peripheral terminal TRPV1, may also become sensitized by the persistent activation of the PKRs due to the presence of PROK2. PC1/PC7 reducing PROK2 availability and blocking the PKRs prevents nociceptor sensitization.

### 3.5. Cytokines in the Sciatic Nerve

Injury of the peripheral nervous system induces immune and nonimmune cells to produce cytokines at and distal to lesion sites. Proinflammatory cytokines contribute to axonal damage and they also stimulate spontaneous nociceptor activity [23].

We and others have analyzed the time-course of the production of pro- and anti-inflammatory cytokines in the nervous tissues after sciatic nerve injury [24, 25]. Schwann cells, resident activated macrophages, and fibroblast rapidly upregulate the expression and production of TNF*α*, IL-1*β*, and G-CSF which are detected between 5 and 10 h after injury. Inflammatory cytokines and chemokines advance the recruitment of blood-borne macrophages that begins 2 to 3 days after the injury concomitantly with production and secretion of IL-6 and IL-10 proteins. IL-17 positive cells have been demonstrated in the endoneurium of the injured sciatic nerve 7 days following CCI [26] and IL-17 has been demonstrated to have a role in later phases of the processes of the development of neuropathic pain [27]. As shown in Table 1, in our experimental setting, at 10 dpi, we found still high levels of IL-1*β*, IL-6, and IL-17 but they were reduced at levels nonsignificantly different from basal in animals treated with the PKR antagonist. Moreover the pharmacological treatment increased the levels of the anti-inflammatory cytokines IL-10, reestablishing the physiological balance between pro- and anti-inflammatory cytokines and bringing the innate-immune response to conclusion far earlier than what happens during spontaneous course of Wallerian degeneration. These data suggest that PC1 is able to direct the polarization of recruited macrophages towards the M2 phenotype [28] which is involved in tissue repair.

### 3.6. Skin Innervation and Thickness

**Figure 10:**
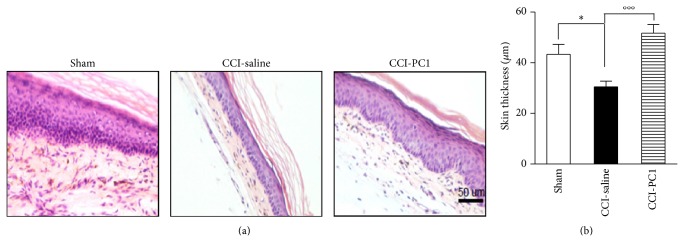
(a) Histological examination of the plantar skin from sham, CCI-saline, and CCI-PC1 mice stained with hematoxylin-eosin at 10 dpi. (b) Quantification of epidermal thickness of the sham, CCI/saline, and CCI/PC1 mice (3 section/animal). Data are expressed as mean ± SEM of 4-5 animals. One-way ANOVA was used for statistical evaluation, followed by Tukey test for multiple comparisons: ^*^ *P* < 0.05; °°°*P* < 0.001.

CCI of the sciatic nerve produces partial denervation of the paw skin and results in a significant reduction in epidermal thickness of the plantar surface of the injured paw [29]. Accordingly, in our setting, epidermis of the paw ipsilateral to the injured nerve was significantly thinner than that of contralateral paw (32.1 ± 2.1 *μ*m versus 44.1 ± 3.3 *μ*m, *P* < 0.05) at 10 dpi. The overall organization of denervated epidermis preserved normal histological organization, but the vital layers showed reduction in thickness. PC1 treatment prevented the thinning of the plantar skin (57.3 ± 2.4 *μ*m) (Figure 10).

**Figure 11:**
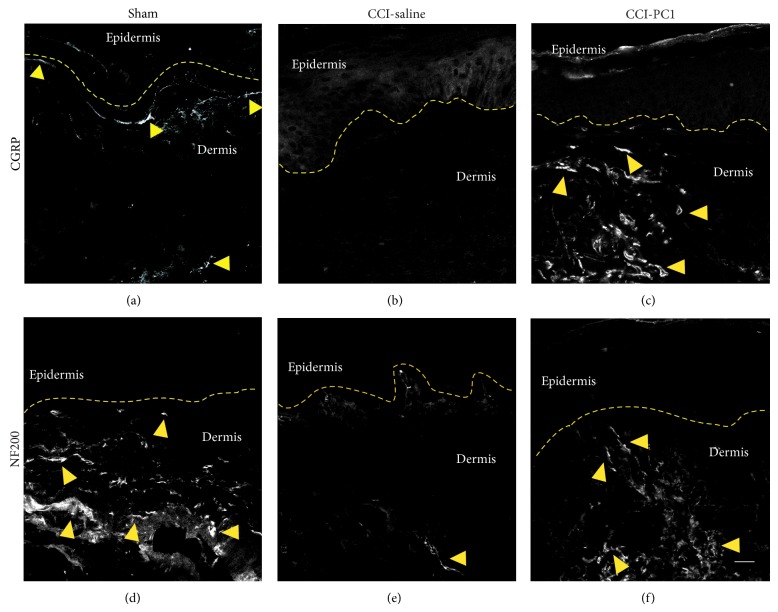
Confocal images of representative skin sections immunostained for CGRP and NF200 from sham-operated, CCI-saline, and CCI-PC1 mice at 10 dpi. (a) In sham-operated mice the CGRP positive fibers were distributed along the dermoepidermal junction. (b) In CCI-saline mice the CGRP positive fibers were absent. (c) In CCI-PC1 mice the CGRP positive fibers were present in dermis. (d) In sham-operated mice the NF200 positive fibers were distributed along the dermoepidermal junction and in dermis. (e) In CCI-saline mice very few NF200 positive fibers were observed. (f) In CCI-PC1 mice the NF200 positive fibers were present in dermis. Dashed line represents the dermoepidermal junction. Scale bar 20 μm.

According to data reported by Peleshok and Ribeiro-da-Silva [30] immunohistochemical staining for CGRP and NF200 of plantar skin of neuropathic mice at 10 dpi demonstrated a virtual absence of CGRP-IR fibres and a dramatic reduction of NF100-IR fibres (Figures 11(b) and 11(e)) in comparison with sham-operated mice (Figures 11(a) and 11(d)). Conversely in PC1-treated neuropathic mice a thick network of CGRP-IR fibres of higher density than that of sham animals was seen and the NF200 innervation pattern resembled that seen in the sham-operated mice also if the signal appeared less intense (Figures 11(c) and 11(f)). These results indicate that repeated treatment with PKR_1_-antagonist protected from axonal damage.

### 3.7. Intraneural Oedema and Blood Nerve Barrier Permeabilization

**Figure 12:**
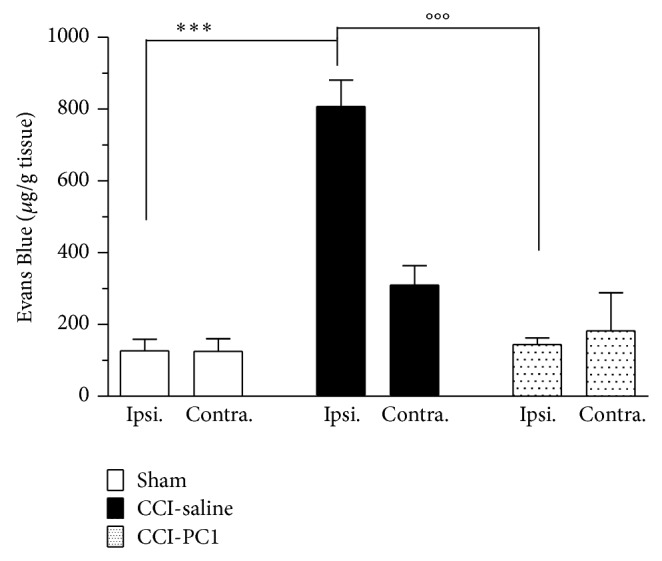
Evans Blue extravasation was measured at 3 dpi in injured and contralateral sciatic nerves. A significant increase in Evans Blue accumulation was evident in the injured nerve. The level of Evans Blue in the contralateral nerve was not significantly different from the level found in sham animals. Evans blue extravasation in the sciatic nerves of mice treated with PC1 (150 μg Kg^−1^, twice/day, at 1 and 2 dpi) did not differ from sham mice. Data are expressed as mean ± SEM of 4-5 animals. One-way ANOVA was used for statistical evaluation, followed by Tukey test for multiple comparisons: ^***^ *P* < 0.001 CCI-saline versus sham mice; °°°*P* < 0.001 CCI-PC1 versus CCI-saline mice.

The constrictive ligatures around the sciatic nerve evoke intraneural edema that causes the nerve to strangulate beneath the ligatures and induce axotomy of mostly large-diameter myelinated axons, sparing mainly C-fibres. Peripheral nerve injury and C-fibres activation increase the blood-nerve barrier (BNB) permeabilization in 24–48 h [31]. The PKRs are expressed also on endothelial cells where they may regulate cell proliferation and vascular leakage [32]. Because we know that systemic injection of PC1 rapidly reduced oedema of the inflamed paw [3] here we decided to verify if the treatment with PKR-antagonist might reduce the blood-nerve barrier permealization consequent to peripheral nerve injury. As demonstrated by Figure 12, the Evans Blue dye extravasation in the sciatic nerve is dramatically reduced already after 2-day-treatment of neuropathic mice with 150 *μ*g kg^−1^ of PC1. Plasma extravasation depends on activation of endothelial PKR_2_ [32]. PC1, at the doses here used in therapeutic schedule, may block also PKR_2_ so exerting further beneficial effect in maintaining the blood-nerve barrier function and in reducing oedema.

Reduced intraneural oedema may result in sparing more axons from degeneration as demonstrated by the fact that skin innervation and skin thickness of neuropathic mice treated with the PKR-antagonist look like that of sham mice.

## 4. Conclusions

The results that we here present support an important role for PROK2 in the pathogenesis of neuroinflammation and neuropathic pain. In support of this hypothesis, we show that blocking the prokineticin receptors with two PKR_1_-preferring antagonists reduced pain and nerve damage. The major finding of this study is that the nerve damage induces an important PROK2-increase in resident and infiltrating cells but also in the sensory neurons and that impairment of PROK2 production is linked to amelioration of pathology indicating PROK2 as precocious determinant in the process of neuroinflammation.

A few studies focused on the mechanisms involved in regulating the expression of PROK2 in myeloid cells demonstrated that PROK2-upregulation occurs through G-CSF-induced activation of STAT3 that binds the enhancer site of its promoter [33, 34], IL-6 cooperates with G-CSF to increase the expression of Bv8/PROK2 gene in neutrophils [35], and moreover Bv8 itself activates STAT3 [36]. Therefore, PROK2 is involved in a self-perpetuating cycle because it also increases its own production as a result of PKR_1_ activation [3].

Intracellular pathway regulating the expression of PROK2 in neurons has not yet been clarified but it may be well-funded to imagine a mechanism like that described in myeloid cells. Indeed, STAT3 activation by G-CSF, IL-6, and IL-1*β* signalling was recently demonstrated in DRG neurons [37, 38]. Receptors and signalling mediators of G-CSF are functionally expressed on sensory neurons also containing TRPV1 [37], some of which constitutively express PROK2 mRNA [11], and G-CSF rapidly produced phosphorylation of STAT3 in cultured DRG neurons [39]. Hence it is likely that the cytokines released early during the Wallerian-degeneration trigger synthesis of PROK2 in the sensitive neurons. STAT3 is required for T helper type 17 (Th17) generation [40] and Ferrara's group recently demonstrated that IL-17 indirectly contributes to recruitment of Bv8-positive granulocytes in tissues infiltrated by Th17 cells [41]. Interestingly, we also demonstrated that switching off the PROK2 production and signalling significantly decreases the burden of proinflammatory cytokines in the lesioned nerve and prompts an anti-inflammatory repair program.

Taken together, these considerations indicate that availability of molecules, like these PKR_1_-preferring antagonists, which in addition to direct modulating nociceptor excitability also control the PROK2 synthesis and release, improves the efficacy in reducing neuroinflammation and neuropathic pain.

## Figures and Tables

**Table 1:** Sciatic nerve cytokine levels ten days after CCI and after seven-day PC-1 treatment.

Cytokine pg/mg protein	Sham	CCI-Saline	CCI-PC1
TNF*α*	0	6.4 ± 3.1	0.97 ± 1.33°
IL-1*β*	64.26 ± 13.59	557.7 ± 121^**^	222.5 ± 110.31°
IL-6	4.92 ± 3.37	13.35 ± 6.15^*^	4.30 ± 2.88°
IL-17	0.97 ± 0.13	13.9 ± 1^*^	3.1 ± 1.8°
IL-10	556.8 ± 50.35	326.8 ± 8.99^*^	867.3 ± 95.2°°°

Values are means ± SD of 5 nerves.

^*^ *P* < 0.05; ^**^ *P* < 0.01 CCI-Saline versus Sham; °*P* < 0.05, °°°*P* < 0.001 CCI-PC1 versus CCI-Saline.
